# Screening of SIRT6 inhibitors and activators: A novel activator has an impact on breast cancer cells

**DOI:** 10.1016/j.biopha.2021.111452

**Published:** 2021-03-05

**Authors:** Jonna Tenhunen, Tomáš Kučera, Marjo Huovinen, Jenni Küblbeck, Egils Bisenieks, Brigita Vigante, Zaiga Ogle, Gunars Duburs, Martin Doležal, Ruin Moaddel, Maija Lahtela-Kakkonen, Minna Rahnasto-Rilla

**Affiliations:** a School of Pharmacy, University of Eastern Finland, Kuopio, Finland; b Faculty of Pharmacy in Hradec Králové, Charles University, Prague, Czech Republic; c Latvian Institute of Organic Synthesis, Riga, Latvia; d Biomedical Research Center, National Institute on Aging, National Institutes of Health, Baltimore, MD, United States

**Keywords:** Activator, Breast cancer, Inhibitor, Sirtuin 6, Virtual screening

## Abstract

Sirtuin 6 (SIRT6), a member of sirtuin family (SIRT1–7), regulates a variety of cellular processes involved in aging, metabolism, and cancer. Dysregulation of SIRT6 is widely observed in different breast cancer subtypes; however, the role and function of SIRT6 in cancer development remain largely unexplored. The aim of this study was to identify novel compounds targeting SIRT6 which may provide a new approach in development of anti-cancer therapy for breast cancer. Virtual screening was utilized to discover potential compounds targeting SIRT6 for in vitro screening. In addition, novel 1,4-dihydropyridine derivatives were synthetized and further subjected for the screening. The impact of the compounds on the deacetylation activity of SIRT6 was determined with HPLC method. The anti-cancer activities were screened for a panel of breast cancer cells. A set of 1,4-dihydropyridine derivatives was identified as SIRT6 inhibitors. A SIRT6 activating compound, (2,4-dihydroxy-phenyl)–2-oxoethyl 2-(3-methyl-4-oxo-2-phenyl-4H-chromen-8-yl)acetate (later called as 4H-chromen), was discovered and it provided 30–40-fold maximal activation. 4H-chromen was proposed to bind similarly to quercetin and place to previously reported SIRT6 activator sites. 4H-chromen was investigated in various breast cancer cells, and it decreased cell proliferation in all cells as well as arrested cell cycle in triple negative cells. Overall, this study describes a highly potent SIRT6 activator and new inhibitors that represent a novel tool to study the mechanism of SIRT6 function.

## 1. Introduction

Human sirtuin 6 (SIRT6), a member of sirtuin family, deacetylases the specific acetylated lysines on histone 3 (H3). This function has an effect on chromatin compaction, transcriptional repression, and DNA damage responses involved in aging, metabolism, and cancer [1,2]. Besides histone deacetylation, SIRT6 has multiple enzymatic activities on various substrates [3–5]. However, SIRT6 seems to be more specific concerning its substrates than other sirtuins, because so far only a few histone deacetylation sites and non-histone substrates have been determined [6]. Nicotinamide adenine dinucleotide (NAD^+^) is a pivotal cofactor for SIRT6 and other sirtuins. Together NAD^+^ and SIRT6 maintain metabolic homeostasis as master regulators of gluconeogenesis and glycolysis. Previously, it has been demonstrated that SIRT6 deficiency induces glucose uptake and glycolysis [7]. SIRT6 deficiency is also linked to cancer although the role in cancer is controversial since SIRT6 can act as tumor promoter or suppressor depending on cell type [6,8].

Depending on pathological processes the deacetylation activity of SIRT6 should be either inhibited or activated. SIRT6 is overexpressed in some cancers and SIRT6 inhibition may have tumor suppressive effects in these cases [9], but also SIRT6 activation can induce anti-cancer activities in SIRT6 deficient cancers. SIRT6 can be regulated by controlling the expression of SIRT6, the amount of cellular NAD^+^, or applying inhibitors and activators. Cellular NAD^+^ is the most important regulator of sirtuin activity since despite of the sufficient tissue levels of NAD^+^, it is the rate-limiting reagent for sirtuin activity [10].

Small molecules can modulate SIRT6 activity, and many natural biochemicals have been reported as SIRT6 regulators [3,11,12]. Some of them such as quercetin and catechin derivatives have showed moderate inhibition against SIRT6 whereas anthocyanidins have shown activation of SIRT6. Recently, we demonstrated that galloflavin and ellagic acid induce SIRT6 activity and increase the deacetylation of lysine 9 on H3 (H3K9) peptide substrate by 40-fold maximal level [13]. Various other molecules have also been shown to modulate SIRT6’s activity [3,14,15], including allosteric activators that stimulate SIRT6 deacetylation through the improvement of turnover rate of NAD^+^ cofactor [15,16], and inhibitors that compete with the substrate and/or the cofactor [17–23].

Here we report a quercetin derivative bearing a chromene moiety as a potent SIRT6 activator, and a new scaffold for developing novel SIRT6 inhibitors. Based on a virtual screening campaign a set of compounds were tested in vitro against SIRT6 and as a result a quercetin-related compound showed an increase in deacetylase activity of SIRT6 and produced 30–40-fold maximal activation. It also dramatically decreased the K_m_ value of H3 acetylated lysine 9 (H3K9Ac) peptide substrate, but not the K_m_ value of NAD^+^. The compound proceeded for testing on a set of various breast cancer cell lines. The outcomes from cell lines indicated diverse cell-line dependent effects.

## 2. Materials and methods

### 2.1. Materials

The compounds 1–6 were purchased from MolPort Inc. Breast cancer cell lines T47-D, ZR-75–1, SKBR3, HCC-1937 and Hs578T were a kind gift from Dr. Maria Berdasco Mennendez for this study. Acetylated histone (H3K9Ac) peptide was from AnaSpec (USA). Fetal bovine serum (FBS) was from Life Technologies (UK). NAD^+^, propidium iodide (PI), and Roswell Park Memorial Institute (RPMI)-1640 medium were from Sigma-Aldrich (USA). Dulbecco modified Eagle medium (DMEM) and non-essential amino acids were from Lonza (Belgium). Digitonin (Calbiochem: 300410) was from Millipore (USA). SIRT6 primers (Hs00966002 m1), RevertAid RT Reverse Transcription Kit (K1691), TaqMan^™^ Gene Expression Master Mix (4369016), and RNase A (EN0531) were from Thermo Fisher Scientific (Germany). Enhanced chemiluminescence (ECL) prime western blotting detection reagents were from Amersham BioSciences (UK). Penicillin/Streptomycin was from EuroClone (Italy). L-Glutamine was from Biowest (France). EZNA total RNA kit (OMEGR6834–02) was from Omega Bio-tek (USA).

The human SIRT6 expression vector hSIRT6-pGEX-6P3 was kindly provided by Prof. Katrin Chua (Stanford, USA). Recombinant glutathione-S-transferase (GST)-tagged SIRT6 was produced by fermentation in *E. coli* BL21(DE3)-pRARE at + 16 °C with 0.1 mM isopropyl β-d-1-thiogalactopyranoside (IPTG) for 20 h and the soluble overexpressed protein was purified on glutathione agarose (Sigma, USA).

#### 2.1.1. Statistic

In all experiments, statistical significance of treated groups to DMSO control groups were analyzed with one-way ANOVA followed by Bonferroni and Dunnett post hoc test, which were all done by using Graph Pad Prism Software version 6 (California, USA).

### 2.2. Ligand and protein preparation

A part of Enamine (4,103,115 molecules) and Chembridge (1,022,400 molecules) databases were downloaded for molecular docking to screen compounds. In addition, a small library of 1,4-dihydropyridine derivatives (approximately 100 molecules) was prepared for the screening. Ligands were prepared in pH 7.0 ± 2.0 with Maestro’s LigPrep module (Schrödinger Release 2014–1, Maestro; Schrödinger Release, LigPrep) and tautomers were generated. The ligands were optimized with OPLS_2005 [24] force field. The ligands of Enamine database were prefiltered with Canvas (Schrödinger Release 2014–1, Canvas) with criteria: Mw less than 700 Da, LogP less than 7, hydrogen bond acceptor (HBA) less than 15, and hydrogen bond donor (HBD) less than 15. The prefiltration resulted in 4,094,462 structures. The ligands from Chembridge database were used after geometry optimization without prefiltering.

SIRT6 contains 355 amino acids and it consists of a catalytic core of 275 residues and N- and C-terminals. SIRT6 has two domains: a Rossmann fold for NAD^+^ binding and a domain containing a zinc-binding motif. Between these two domains is a cleft where the substrate and various regulators can bind. The structure of SIRT6 (PDB ID:3K35, resolution 2 Å) [25] was used for virtual screening campaign. The protein structure was prepared using Protonate 3D in Molecular operating environment, MOE (MOE 2013) with pH of 7.0, temperature of 300 K and salt concentration of 0.1 mol/l. The quality of the prepared protein structure was checked with Ramachandran plot.

### 2.3. Virtual screening campaign

Molecular operating environment, MOE (MOE 2013) was employed for identifying hits with pharmacophore model (Fig. S1), similarity search, and molecular docking. Pharmacophore module was used to generate pharmacophore models which were used as a 3D structural query for retrieving potent hits from Enamine and Chembridge databases. Similarity search module of MOE was also used to search hits from the databases.

The nicotinamide (NAM)-moiety pocket in SIRT6 was defined as the pocket for molecular docking. Docking was performed twice with MOE-Dock algorithm using MMFF94x force field [26]. At first docking round Triangle matcher was used as placement method and London dG function was used for scoring. The best scored compounds were redocked flexibly by allowing bond rotation. Triangle Matcher was used as placement method, first rescoring function was London dG, refinement method was Forcefield, and second rescoring function was GBVI/WSA dG. The molecular docking results were visually inspected, and a set of hits was redocked with Glide (Schrödinger Release 2014–1, Glide) to select hits for in vitro studies. In both methods, the grid box was defined with residues Arg65, Asp116, and His133, and default settings and standard precision (SP) were used.

### 2.4. Molecular docking of validated hits

Schrödinger Maestro (Schrödinger Release 2019–4, Maestro) was used in investigating the identified SIRT6 regulators. SIRT6 structure in complex with adenosine diphosphate ribose (ADPr) and quercetin (PDB ID: 6QCD, resolution 1.84 Å) [15] was prepared at pH of 7.4 with Protein Preparation Wizard (Schrödinger Release 2019–4, Protein Preparation Wizard). Briefly, missing side chains and loops were filled in with Prime (Schrödinger Release 2019–4, Prime). Triethylene glycol molecules, ethanediol molecules, and sulfate ions were removed from the structure. Ionization states for zinc ion, ADPr, and quercetin were generated. H-bonds were assigned with PROPKA [27] and waters were removed. Structure was minimized at OLPS3e force field [28] using RMSD of 3.0 Å for heavy atom converge. Ligand structures were built with Maestro 2D Sketcher tool and prepared with LigPrep (Schrödinger Release 2019–4, LigPrep). Possible ionization stages at target pH 7.4 were generated with Epik (Schrödinger Release 2019–4, Epik) as well as possible stereoisomers.

Glide was used for docking the validated hits (Schrödinger Release 2019–4, Glide). The grid center for activator was set according to the co-crystallized quercetin [15], and the docking position was restricted to the position of quercetin with maximum tolerance of 1.0 Å. The grid for inhibitors was created close to the binding site of NAM-moiety. The grid center situated in the middle of residues Arg65, Asn114, Val115, and Asp116. The activator was docked with standard precision (SP) and the inhibitors were docked with extra precision (XP) method. Default settings were applied in both activator and inhibitor dockings: van der Waals radii scaling for the ligand nonpolar parts was applied with scaling factor of 0.8 and with partial charge cutoff of 0.15. Ligand structures were docked flexibly by sampling nitrogen inversions and ring conformations. Sampling of torsions was biased only for amides.

### 2.5. HPLC assay

SIRT6 HPLC assay was performed as previously reported [11,12]. Briefly, compounds in DMSO or DMSO (control) were incubated for 30 min with GST-SIRT6 (3 μg/well), H3K9Ac (70/200 μM) and 500 μM NAD^+^ in Tris-HCl Buffer (25 mM, pH 8.0) at + 37 °C. In the case of a steady-state kinetic analyses increasing concentration either NAD^+^ or H3K9Ac with saturating concentration of H3K9Ac (3 mM) or NAD^+^ substrates (2.5 mM) were used. Control samples for compounds without NAD^+^ or SIRT6 were carried out. The deacetylation reaction was terminated by adding 6 μL of cold 10% formic acid and centrifuged for 15 min. The samples were analyzed by reversed-phase HPLC. The formation of deacetylated product (H3K9) and substrate (H3K9Ac) peaks was monitored and subsequently quantified by measuring area under the curve. Statistical analysis was carried out using Graph Pad Prism Software version 6 (California, USA).

### 2.6. SIRT1–2 in vitro assays

The method is described in the BioMol product sheet (Enzo Life Sciences, Ann Arbor, MI, USA). BioMol KI177 substrate for SIRT1 and KI179 substrate for SIRT2 were used. Briefly, the reaction was started by incubating the enzyme (SIRT1, SIRT2) with the reaction mixture containing acetylated peptide substrate (0.7 K_m_: 58 μM for SIRT1, 198 μM for SIRT2), NAD^+^ (0.9 K_m_: 558 μM for SIRT1, 547 μM for SIRT2), and DMSO or compound in DMSO (5% final DMSO concentration). Samples were incubated for 1 h at 37 °C, thereafter the developer and NAM (2 mM) were added, and the incubation was continued for 45 min at 37 °C. The fluorescence was determined with excitation and emission wavelengths of 355 nm and 460 nm, respectively, using EnVision2104 Multilabel Reader (PerkinElmer, Waltham, MA, USA).

### 2.7. Myristoyl assay

The method is described in the BPS Bioscience product sheet (San Diego, CA, USA). Briefly, the reaction was started by incubating the SIRT6 enzyme with the reaction mixture containing myristoyl peptide substrate (10 μM), NAD^+^ (500 μM) and DMSO or compound in DMSO (3% final DMSO concentration). NAM was used as a control compound. Samples were incubated for 30 min at 37 °C, thereafter the developer and NAM (2 mM) were added, and the incubation was continued for 15 min at 37 °C. The fluorescence was determined with excitation and emission wavelengths of 355 nm and 460 nm, respectively, using EnVision2104 Multilabel Reader (PerkinElmer, Waltham, MA, USA).

### 2.8. Cell culture and cell cultural assays

#### 2.8.1. Cell culture

Different breast cancer cells were cultured in growth medium (Table S1), at + 37 °C (5% CO_2_ + air) for 14 days before the treatments. After 24 h (for cell viability and number, PCR) and 6 h (cell cycle analysis), cells were treated with 0.5% DMSO (control) or various concentrations of compound 1 for 24 h.

#### 2.8.2. Reverse transcription-polymerase chain reaction (PCR)

After the 24 h chemical treatment, the treatment medium was aspirated, and the cells were washed with 1xD-PBS. Total RNA was extracted and purified using EZNA® Total RNA I kit according to manufacturer’s instructions. The RNA concentration was determined by UV absorbance at 260 nm and 280 nm, and 500 ng of purified RNA was reverse transcribed (RevertAid) to cDNA using random priming. The expression of SIRT6 was determined from the samples (n = 3), using real-time quantitative PCR (RT-qPCR) with TaqMan gene expression assays and QuantStudio 5. β-actin was used as an endogenous control. Relative quantification of gene expression was performed using the ΔΔCt method assuming equal amplification efficiencies with QGene program (Eq. 2 [29]).

#### 2.8.3. Cell viability and number

PI-digitonin cell viability measurement was carried out as described previously [30]. This method is based on the fluorescence of PI that can only enter cells and nuclei with damaged membranes, while the membranes of viable cells are impermeable to PI. Briefly, cells were exposed to compound 1 on 48-well plates for 24 h and followed by addition of PI to each well. The fluorescence was measured after 20 min at the excitation wavelength of 531 nm and the emission wavelength of 615 nm using Victor (Wallac, 1420 Multilabel Counter). In order to obtain the maximal fluorescence value, which reflects the total number of cells, the cells were incubated with digitonin, which damage the cell wall and the nuclear membrane, making them permeable to PI. Maximal fluorescence was measured after 20 min at the excitation wavelength of 531 nm and the emission wavelength of 615 nm.

#### 2.8.4. Cell cycle analysis by flow cytometry

In order to perform the cell cycle analysis, cells were seeded on 6-well plates and collected after 24 h treatment with compound 1 (n = 3). Cells were fixed in ice-cold ethanol/PBS (70:30) overnight at − 20 °C followed by resuspension with PBS containing 0.15 mg/mL RNase and incubation at 50 °C for 1 h. The DNA content was analyzed by staining the cells with PI (20 μg/mL) and the distribution of the cells in different cell cycle phases was measured with a Novocyte Quanteon flow cytometer (ACEA Biosciences). Data are presented as mean values of three independent experiments.

## 3. Results

### 3.1. Chemistry

**Scheme 1.**
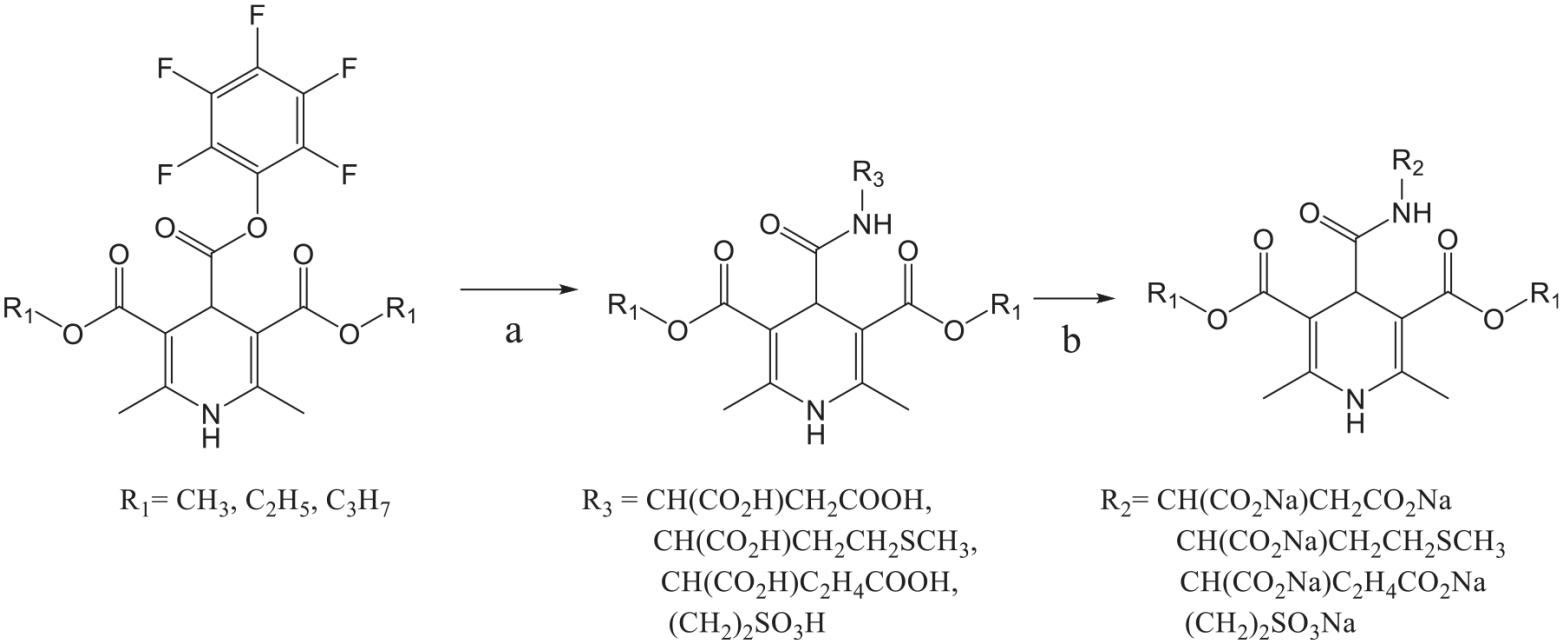
Preparation of 1,4-dihydroisonicotinic acid derivatives 7–10. Reagents and conditions: a) appropriate amino acid, dimethylformamide (DMF), Et_3_N, 0 °C, 10 min, rt, 48 h; b) EtOH, 1 N NaOH water sol., rt, stirred continuously, precipitated with acetone.

Compounds 7–10 are derivatives of 1,4-dihydroisonicotinoyl acid (Table S2) and belong to the group of typical dihydropyridine derivatives – quasi- dipeptides or peptidomimetics [31,32] (Table S2). Compounds 7–10 were prepared in a two stage (Scheme 1) procedure that involved the initial synthesis of pentafluorophenyl 2,6-dimethyl-3, 5-diethoxycarbonyl-1,4-dihydroisonicotinate and the further reaction with appropriate amino acid according to the patent [Pat US 4485239A] [33].

### 3.2. Novel regulators were identified by virtual screening

**Fig. 1.**
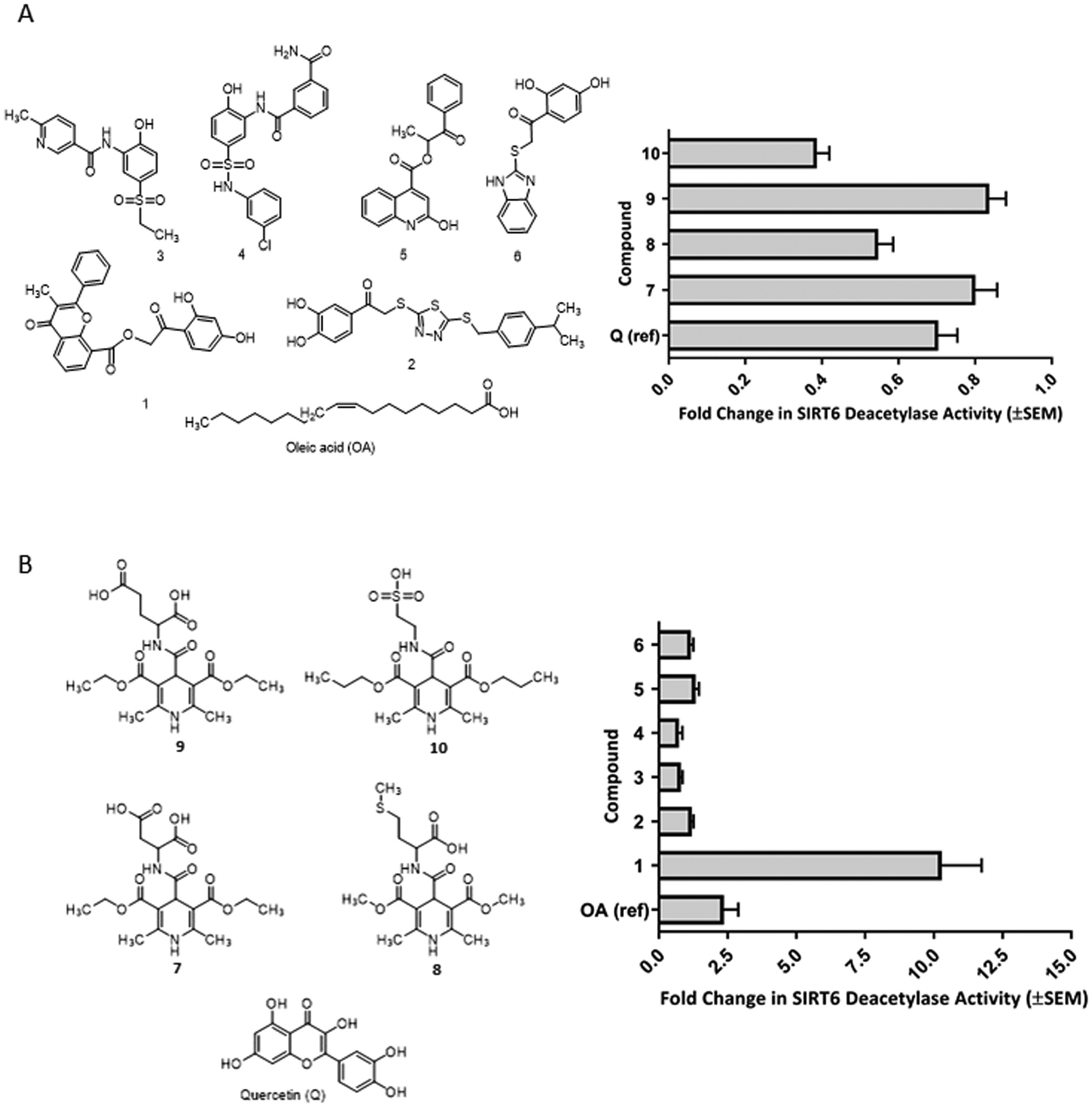
The compounds identified with virtual screening modulate SIRT6 deacetylation activity. (A) Activators (compound 1–6) with oleic acid (OA); (B) inhibitors (compounds 7–10) with quercetin (Q). Compounds were subjected HPLC based assay at 100 μM concentration. The data are presented as mean ± SEM (n = 3).

Three databases were used in the virtual screening campaign: Enamine, Chembridge, and small library of synthetic 1,4-dihydropyridine compounds. Enamine and Chembridge databases were screened with pharmacophore and similarity approaches. The pharmacophore models were generated based on a set of known sirtuin regulating compounds (resveratrol, Ex-527, sirtinol, and quercetin) [12,34–36]. The final pharmacophore model had an aromatic feature, three HBAs and one HBD (Fig. S1). The pharmacophore search resulted in 3362 and 2387 hits from Enamine and Chembridge databases, respectively. The similarity screenings were performed with whole structure and fragments of compounds by Parenti et al. [19]; the screenings resulted together in 15,738 and 8234 hits from Enamine and Chembridge, respectively. All hits from pharmacophore and similarity screening together with the 1,4-dihydropyridine derivatives were first docked to SIRT6 with MOE using fast docking protocol. The scoring was compared to the score of docked quercetin, and 68 pharmacophore hits, 70 similarity search hits, and 10 of 1,4-dihydropyridine derivatives were selected for redocking with MOE using a more accurate protocol. From these redockings 53 hits were selected to be docked with Maestro Glide. Finally, 10 hits were selected for in vitro screening after visual inspection of molecular docking poses (Fig. 1).

### 3.3. Compounds modulated deacetylation activity of SIRT6

The deacetylation activity was tested with fluorogenic based assay (data not shown) and HPLC based assay. Fig. 1 shows SIRT6 activation pattern of known SIRT6 modulators, oleic acid and quercetin, and top ranked compounds at 100 μM concentration in HPLC assay. Oleic acid doubled whereas quercetin inhibited 30% SIRT6 deacetylation activity. Compounds 1–6 showed increase in the deacetylase activity of SIRT6 (Fig. 1A). The structural analog of quercetin, compound 1 (4H-chromen) was the most active compound, producing a 10-fold activation. Instead, structurally diverse compounds 2–6 displayed only weak activation. The selectivity of 4H-chromen was also evaluated against the most widely studied sirtuins, and as a result no inhibition towards SIRT1 was observed, but the compound produced moderate inhibition (53 ± 1.3%) of SIRT2 at 200 μM concentration. Compounds 7–10 (Fig. 1B) share 1,4-dihydropyridine scaffold with various moieties, and they exhibited variable inhibition activity against SIRT6; the most potent 1,4-dihydropyridine, compound 10 displayed about 60% inhibition. A set of variable analogs of 4H-chromen was also tested but they displayed weak activity (Table S3).

**Fig. 2.**
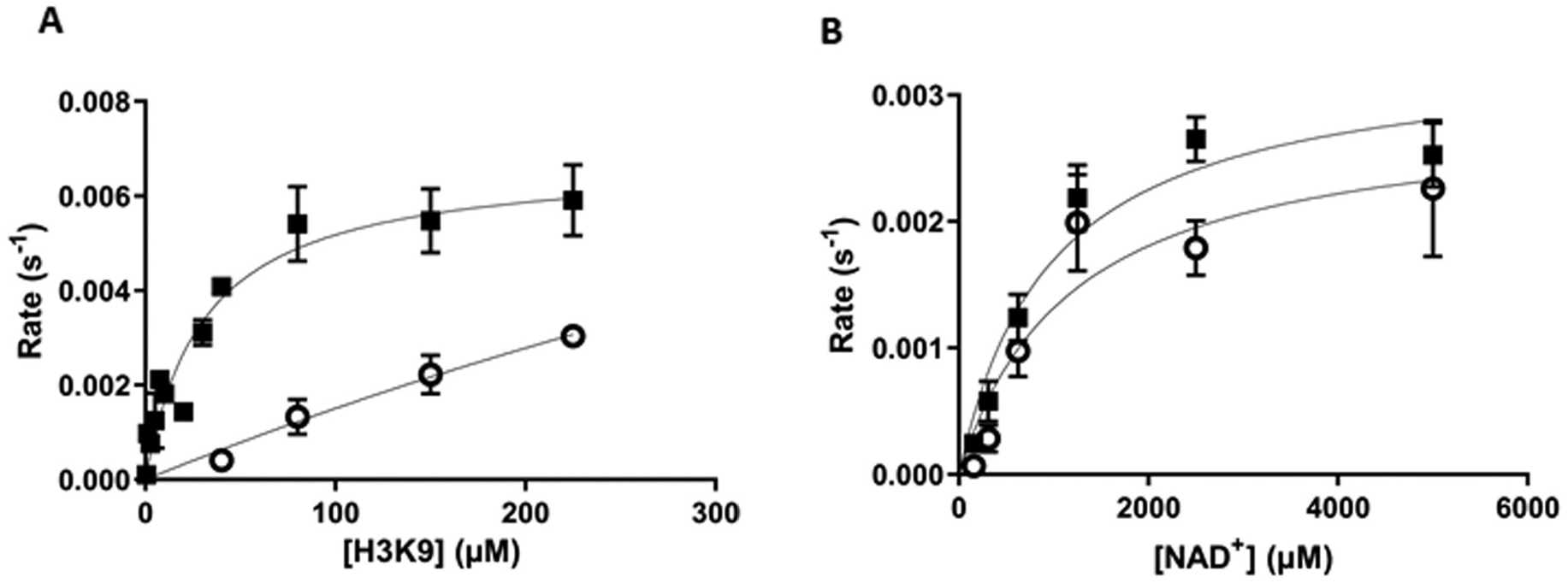
4H-chromen induced a decrease in K_m_ value of acetylated H3K9Ac substrate. Michaelis-Menten analysis of (A) H3K9Ac (0–225 μM) and (B) NAD^+^ with (filled square) and without (unfilled circles) 4H-chromen at 50 μM concentrations. The data are presented as mean ± SEM (n = 3).

4H-chromen provided EC_50_ value of 80 ± 12 μM with 30–40-fold maximal activation (Fig. S2). In order to obtain kinetic parameters, a steady-state kinetic analyses were performed by increasing either the concentration of H3K9Ac peptide or NAD^+^ in the presence or absence of 50 μM concentration of 4H-chromen. The compound dramatically decreased the K_m_ value of H3K9Ac substrate from 1120 ± 580 μM to 31 ± 5.4 μM (Fig. 2A, Table 1). However, similar effect was not observed on the K value of NAD^+^ m : K_m_ values were 970 ± 265 μM and 1190 ± 450 μM with and without 4H-chromen, respectively (Fig. 2B). The compound also improved the catalytic efficiency of H3K9Ac and subsequently increased k_cat_/K_m_ by decreasing the K_m_ value of the substrate (Table 1). However, the compound did not have a similar effect on the kinetical values (k_cat_, k_cat_/K_m_) of NAD^+^.

We also studied the effect of 4H-chromen on SIRT6 demyristoylase activity in the presence of increasing concentration of compounds (Fig. S3). Dose-response study displayed that the compound achieved about 60% maximal inhibition whereas NAM as a control compound showed 86 ± 13% inhibition at 200 μM concentration.

### 3.4. Compounds have different poses in the SIRT6 binding pocket

**Fig. 3.**
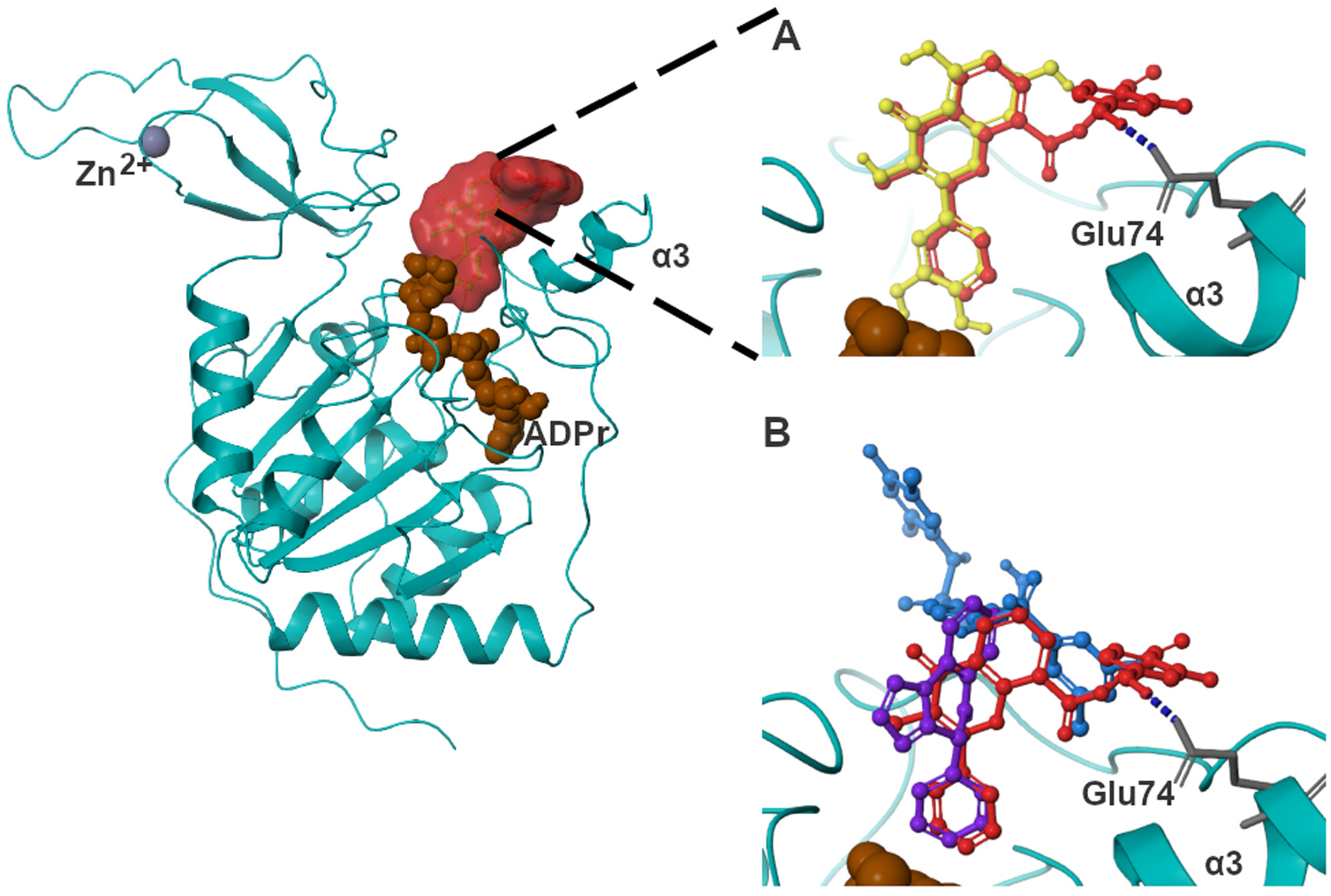
4 H-chromen (red) aligns with co-crystallized quercetin (yellow) (A) and occupies the binding sites of UBCS039 (purple) and MDL-801 (blue) (B). Blue dashed line represents hydrogen bonding.

The interactions of the most active compounds, 4H-chromen, 8, and 10 were examined in more detail. The docking results suggested that when the quercetin-like methylflavone moiety of 4H-chromen is placed according to the crystallized quercetin, the rest of the compound is placed next to α3 helix (Fig. 3A). Investigations of the interactions showed that the resorcinol moiety forms a hydrogen bond with Glu74 located in α3 helix. This pose had similarities to the poses of previously reported SIRT6 activators, UBCS039 [14] and MDL-801 [16] (Fig. 3B).

Inhibiting compounds 8 and 10 showed two alternative poses in the binding pocket. They either occupied the NAM-moiety site of NAD^+^ or alternatively placed partially to the site of acetylated substrate (Fig. S4). In the poses where the NAM-binding area was occupied, π-π interactions and hydrogen bonds with Phe82 and Trp188 were formed. A hydrogen bond with Trp188 was the most common interaction in those poses where the binding site of acetylated substrate was occupied. Additional interactions were formed but they varied between the compounds.

### 3.5. Breast cancer cell lines displayed variation on SIRT6 mRNA levels

**Fig. 4.**
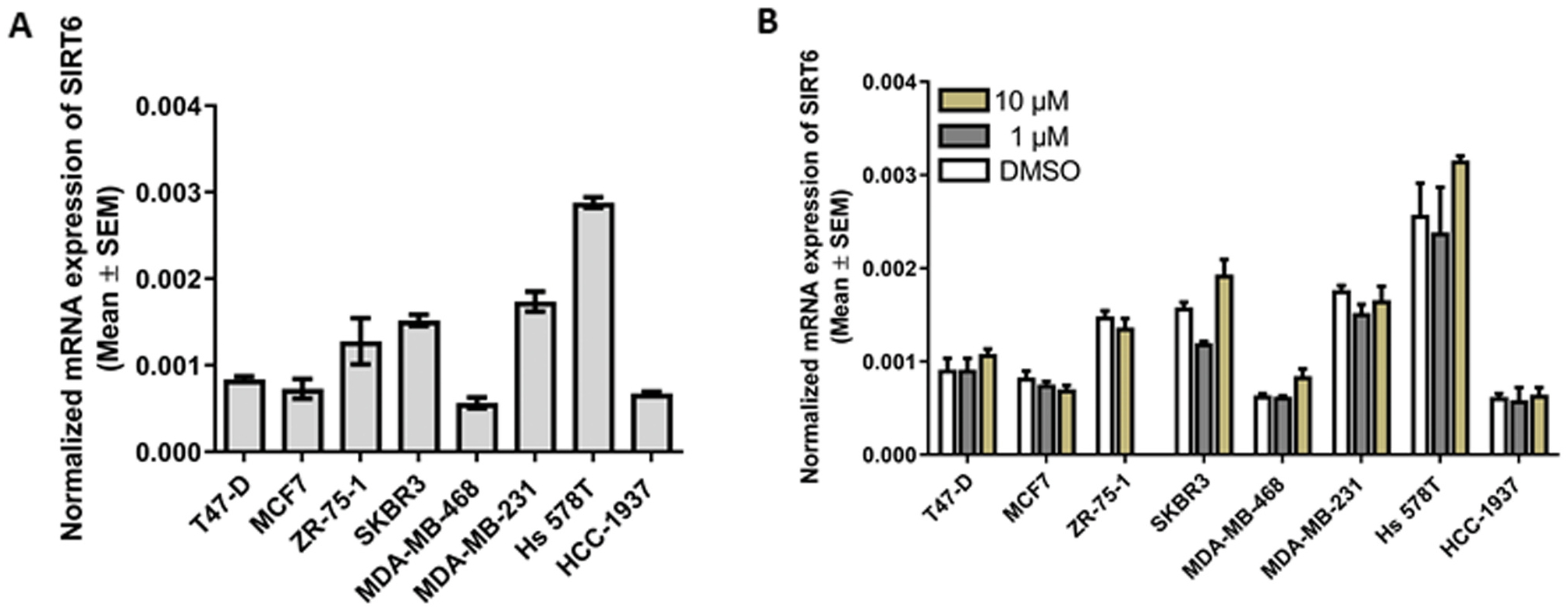
Breast cancer cell transcript data indicates variation on SIRT6 mRNA expression. SIRT6 mRNA expression levels after treatment with (A) medium, and with (B) DMSO control or 4H-chromen. Data represent the mean ± SEM of three experiments.

The SIRT6 gene expression profiles in the different breast cancer cells were investigated. The breast cancer molecular subtypes studied here spanned luminal A (T47-D, MCF7, ZR-75–1), human epidermal growth factor receptor 2 (HER2)^+^ (SKBR3), triple-negative A (MDA-MB-468, HCC-1937), and triple-negative B (MDA-MB-231, Hs578T). The result showed variation on SIRT6 expression between different breast cancer cells lines (Fig. 4A). Triple negative A cell lines showed relatively the lowest levels of SIRT6 mRNA whereas triple negative B displayed the highest levels. Breast cancer cell lines were also treated with vehicle or 4H-chromen and the results indicated that SIRT6 levels remained relatively constant displaying statistically insignificant changes in cell lines (Fig. 4B).

### 3.6. 4H-chromen demonstrated inhibition of cell proliferation and the arrest of cell cycle

**Fig. 5.**
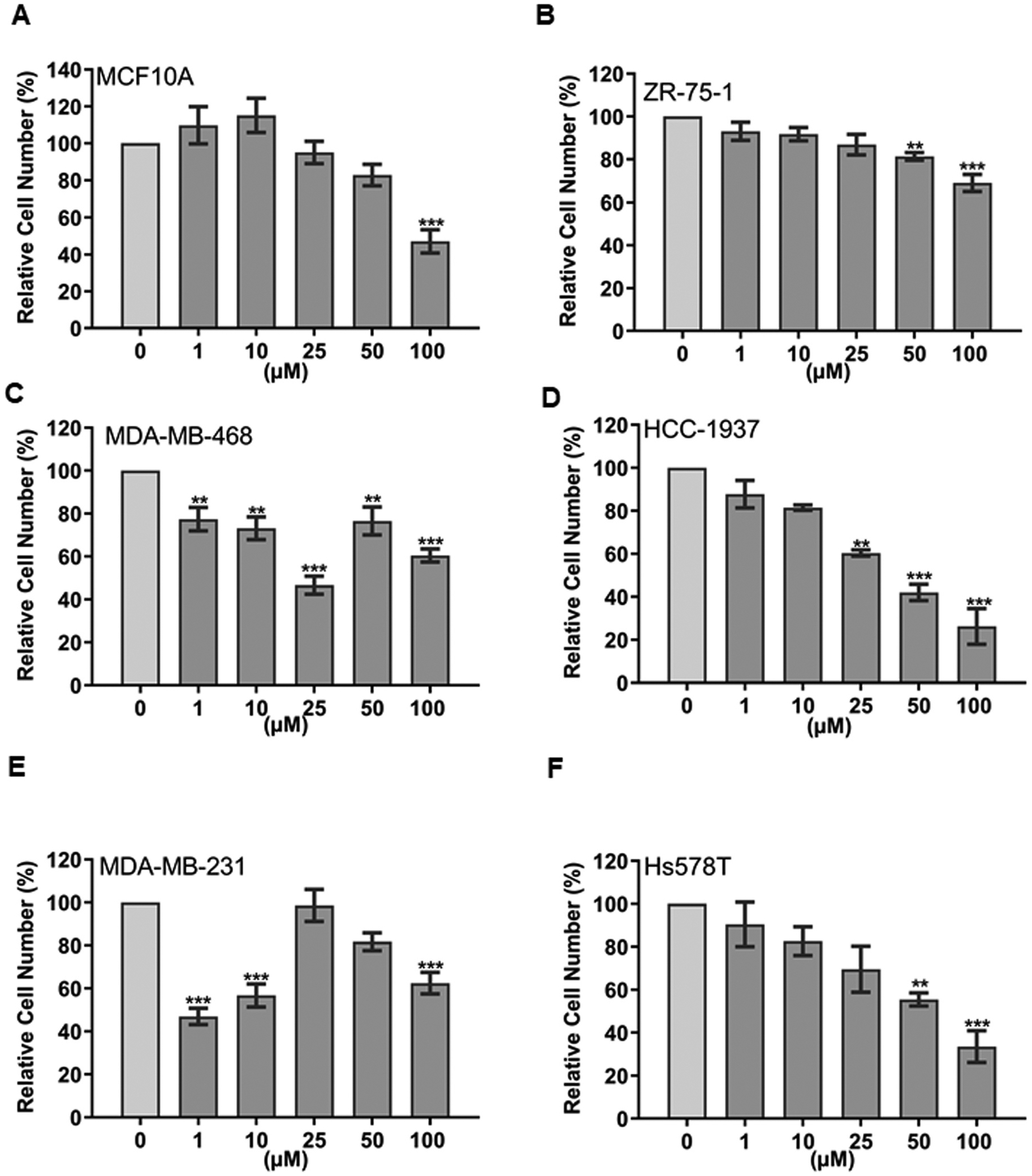
4H-chromen decreased relative cell number in different breast cancer. Cell number is expressed as percent from control. Data represent the mean ± SEM (n = 3), and the statistical analysis was carried out with one way-ANOVA with Dunnett post hoc test by comparing treated groups (gray bars) to DMSO control groups (light gray bars) (**p < 0.01 vs. control and ***p < 0.001 vs. control).

According to the cell viability assays, 4H-chromen has significant effect on viabilities of SKBR3 and Hs578T cell lines at the highest (100 μM) concentration but not on other cell lines (Fig. S5A–F). 4H-chromen decreased the relative cell number of MCF10A cells at 100 μM concentration (Fig. 5A) and dose-dependently ZR-75–1 (Fig. 5B), MCF7 (Fig. S5C), and SKBR3 (Fig. S5D) cells indicating the effect on cell proliferation. In the case of triple negative A cell lines, 4H-chromen displayed significant effect on MDA-MB-468 (Fig. 5C) cells at all concentrations and on HCC-1937 (Fig. 5D) cells at concentration of 25–100 μM. Also, a significant decrease in cell numbers was demonstrated in both triple negative B cell lines (Fig. 5E and F).

**Fig. 6.**
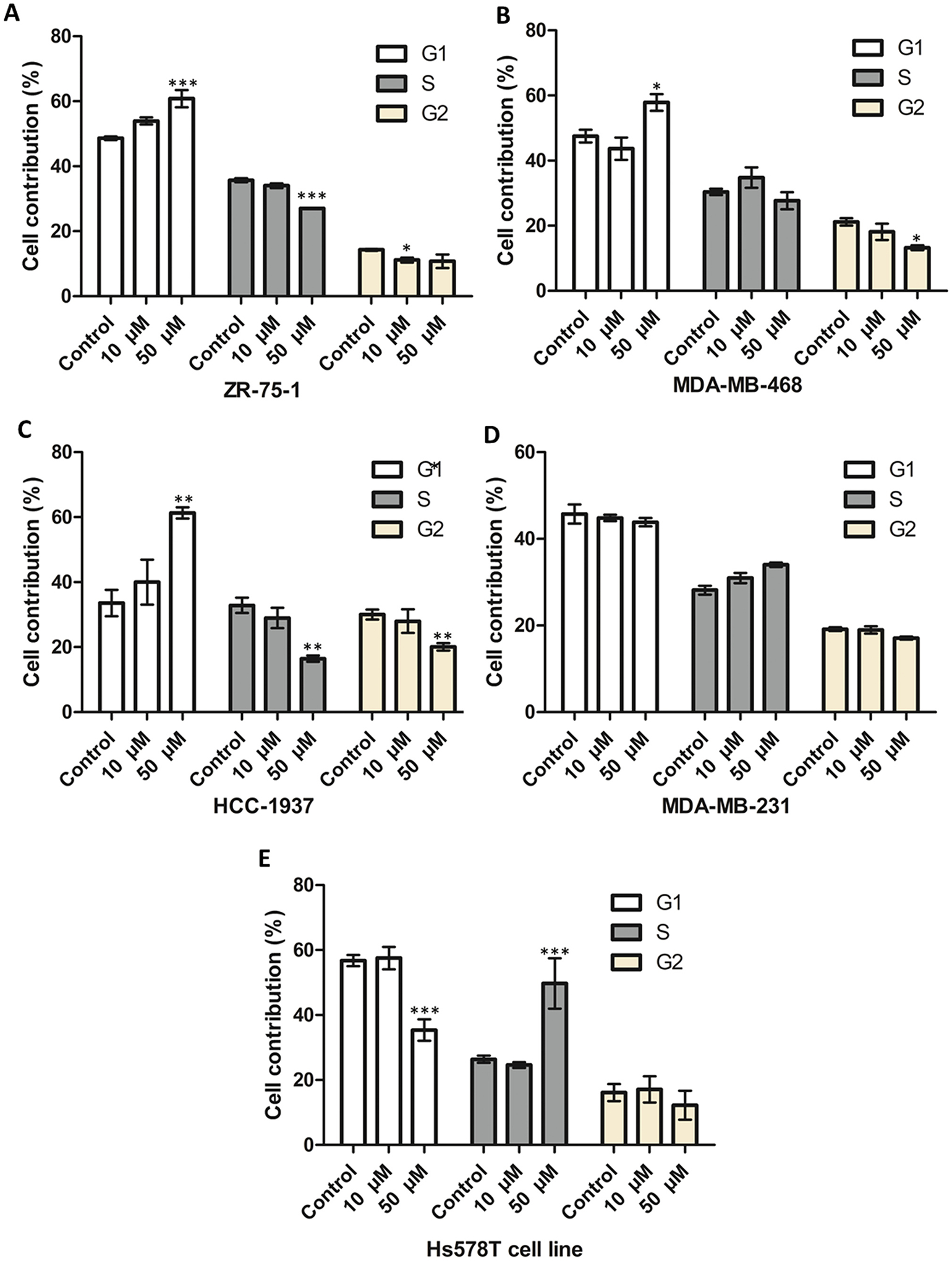
4H-chromen affects cell distribution in different breast cancer. Data represent the mean ± SEM (n = 3), the statistical analysis was carried out with one way-ANOVA with Dunnett post hoc test by comparing treated groups to DMSO control groups (*p < 0.05 vs. control, **p < 0.01 vs. control and ***p < 0.001 vs. control).

The effect of 4H-chromen on cell distribution in different cell cycle phases was further examined in selected cell lines (Fig. 6A–E). In ZR-75–1 (Fig. 6A) and HCC-1937 (Fig. 6C) cell lines 4H-chromen induced significant accumulation of cells in the G1-phase along with a decrease of cells in the S- and G2-phases, compared to the control cells. Similarly, 4H-chromen arrested MDA-MB-468 cells (Fig. 6B) at the G1-phase but significantly decreased cells in the G2-phase. Interestingly, in the case of triple negative B cell lines (Fig. 6D and E), the trend was opposite as the compound caused cell cycle arrest at the S phase although the effect was significant just on Hs578T cell line.

## 4. Discussion

We carried out virtual screening campaign to discover new SIRT6 modulators. They were searched based on the structure and features of known sirtuin modulators [12,19,34–37]. We were able to reduce the enormous chemical space to a manageable number of hits with the potential to bind with SIRT6. Finally, 10 compounds were tested in vitro and three of them modulated SIRT6 catalytic activity.

4H-chromen increased SIRT6 deacetylation activity resulting in 30–40-fold maximal activation of SIRT6. It contains a chromene scaffold and thus, is structurally related to quercetin, a known SIRT6 modulator. Besides quercetin, it is also related to other polyphenolic biochemicals such as anthocyanidins that have been demonstrated to stimulate SIRT6 deacetylation activity [12]. In order to examine structural diversity of 4H-chromen, a set of its analogs were also tested, but they showed only weak activity against SIRT6 (Table S3). The results suggest that the removal of hydroxyl groups in resorcinol moiety reduced dramatically SIRT6 deacetylation activity. Also compounds with halogen groups in resorcinol moiety displayed only minor effect against SIRT6. The substitution of resorcinol moiety with pyrrole or hydrophobic alkyl group decreased activity to minimum.

We identified also that some of compounds with 1,4-dihydropyridine scaffold displayed potent inhibition against SIRT6. Structurally diverse 1,4-dihydropyridine derivatives have been reported to modulate activities of SIRT1 [38,39]. Discovery of inhibitory activity of these compounds against SIRT6 adds additional property for 1,4-dihydropyridine compounds. Besides activity against sirtuins, 1,4-dihydropyridine derivatives display also potential antioxidative activities [39,40] as well as neuroprotective and memory-enhancing properties [31,32] and thus they may have broad therapeutic implications for cancer and other age-related diseases.

SIRT6 has a large hydrophobic pocket between Rossmann fold and Zn-binding domain. This pocket includes the catalytic center of deacetylation reaction. Due to the large size of this pocket, there are different parts where structurally variant regulators might bind and have effect on SIRT6’s function. Based on molecular docking, 4 H-chromen might occupy a part of binding site proposed for SIRT6 activator MDL-801 [16]. However, the quercetin-like moiety placed to the binding site of quercetin [15] and SIRT6 activator UBCS039 [14]. This site overlaps with the proposed binding site of myristoylated substrate and SIRT6 activating fatty acids [3,12,14]. Similar to quercetin [15], UBCS039 [14], and myristic acid [3], 4H-chromen also inhibited slightly the demyristoylase activity of SIRT6, but only with about 60% maximal inhibition. Since maximal inhibition was so low, IC_50_ could not be determined reliably. The mechanism of small molecule activation is still poorly understood, and further studies are needed. Kinetic analysis suggested that 4H-chromen stimulates SIRT6 deacetylation with a similar mechanism that is presented for small molecule activators and myristic acid [41,3]. 4H-chromen increased the k_cat_/K_m_ of H3K9Ac by influencing the K_m_ but not the k_cat_ value of H3K9Ac indicating that the compound increases the affinity of the substrate to the active site of the enzyme. This might indicate that the compound enhances the deacetylation. 4H-chromen does not increase the affinity of NAD^+^. However, some compounds have also been observed to have alternative mechanism of activation [16], but kinetical analyses have been performed for only a few compounds.

In this study we showed variation of SIRT6 mRNA gene expression levels in a panel different breast cancer cells. Triple negative A cells displayed the lowest levels whereas triple negative B showed the highest levels. Both triple negative cells are characterized by the lack of expression of hormone receptors, estrogen receptor alpha (ERα), progesterone (PR) and HER2. However, they have distinct morphologies: triple negative A cells may have either luminal-like or basal-like morphologies and more differentiated subtype than triple negative B cells. Instead, triple negative B cells exhibits claudin-low molecular subtype and show enrichment for markers associated with the epithelial-mesenchymal transition. Thus, they have a more mesenchymal-like appearance and are more likely invasive [42,43]. High levels of SIRT6 promote inactivation of many tumor suppressor proteins such as p53 that is mutated in six out of the eight tested breast cancer cell lines including triple negative cells, whereas low levels of SIRT6 increase the expression of cellular myelocytomatosis (c-MYC) and hypoxia-inducible factor (HIF1), resulting in accelerated cellular proliferation and glycolysis, respectively [43].

4H-chromen showed only minor changes in SIRT6 gene expression levels, however, it decreased cell proliferation in all cell lines. Both downregulation and overexpression of SIRT6 have been reported to affect to the proliferation by arresting cell cycle. 4H-chromen also arrested cell cycle in G1-phase in triple negative A cells but in S/G2-phase in triple negative B cells. In the hepatocellular carcinoma cells SIRT6 overexpression has been reported to arrest cell cycle in the G1-phase which subsequently promote tumor suppressing effect in liver [9]. On the other hand, in human melanoma cells SIRT6 knockdown induced G1-phase arrest and senescence-like phenotypes [44]. The reasons for these controversial observations are not well understood, but one reason might be the complexity of the SIRT6 target proteins. It may also indicate opposite roles of SIRT6 at different stage of tumor progression and in different tumor cell sub-populations. Overall, previous studies showed controversial roles of all SIRTs in breast cancer. SIRT genes were differentially expressed in breast cancer tissues and cancer cell lines [45]. In the case of SIRT6, low SIRT6 expression was associated with poor overall survival in the hormone-receptor positive subtype, however, SIRT6 was reported to be up-regulated in breast cancer tissues but the changes were statistically insignificant [45]. In another study, low expression of SIRT6 predicted poor overall survival in triple-negative breast cancer patients [46].

The unique expression profiles of SIRT6 and other sirtuins may be a characteristic feature in different types of cancers, and different stages of tumorigenesis. It may also influence treatment response in cancer. In order to see the big picture, it would be important to screen changes in SIRT6 levels in many kinds of cancer cell lines which may help to discover safe individual-specific treatments for cancer.

## 5. Conclusions

In this study we presented a highly potent SIRT6 activating compound, 4H-chromen, that is structurally related to quercetin and also a set of SIRT6 inhibitors consisting of 1,4-dihydropyridine scaffold. Modeling studies suggested a putative hydrophobic binding site for 4H-chromen and 1,4-dihydropyridines with favorable interactions with SIRT6. 4H-chromen showed a significant effect on proliferation in all subtypes of breast cancer cell lines and induced also a cell cycle arrest in triple negative cells. As a result, compound may possess anti-cancer activities against different breast cancer cells and may have potential in the development of novel anticancer drugs. We also showed the unique SIRT6 profile in distinct breast cancer cells. Exploring the changes in SIRT6 expression will help discover more efficient and safer individual-specific treatments.

## Supplementary Material

Supplemental Files

## Figures and Tables

**Table 1:** Effects of 4H-chromen on Michaels-Menten kinetical parameters of H3K9Ac substrate and NAD^+^.

	*K*_*m*_ (μM)	*k*_*cat*_ (s-1) *x10*^*−3*^	*K*_*cat*_*/K*_*m*_ (M^−1^s^−1^)
DMSO, _H3K9Ac_	1120 ± 580	1.4 ± 0.9	1.3
4H-Chromen, _H3K9Ac_	31 ± 5.4	5.3 ± 0.4	171
DMSO, _NAD+_	1190 ± 450	2.2 ± 0.4	1.9
4H-Chromen, _NAD+_	970 ± 265	2.6 ± 0.3	2.7

## Abbreviations

ADPr: Adenosine diphosphate ribose
DMEM: Dulbecco modified Eagle medium
DMF: Dimethylformamide
ELC: Enhanced chemiluminescence
ERα: Estrogen receptor alpha
FBS: Fetal bovine serum
GST: Glutathione-S-transferase
HBA: Hydrogen bonding acceptor
HBD: Hydrogen bonding donor
4H-chromen: (2,4-dihydroxy-phenyl)–2-oxoethyl 2-(3-methyl-4-oxo-2-phenyl-4H-chromen-8-yl) acetate
HER2: Human epidermal growth factor receptor 2
HIF1: Hypoxia-inducible factor
H3: Histone 3
H3K9: Lysine 9 on H3
H3K9Ac: Acetylated lysine 9 on H3
IPTG: Isopropyl β-d-1-thiogalacto-pyranoside
c-MYC: Cellular myelocytomatosis
NAD^+^: Nicotinamide adenine dinucleotide
NAM: Nicotinamide
PCR: Polymerase chain reaction
PI: Propidium iodide
PR: Progesterone
RPMI: Roswell Park Memorial Institute
SIRT6: Human Sirtuin 6
SP: Standard precision
