# Supplementary material for: Screening of SIRT6 inhibitors and activators: A novel activator has an impact on breast cancer cells
Source: Biomed Pharmacother. Author manuscript; Available in PMC 2025 Apr 28. (PMC12036750; doi:10.1016/j.biopha.2021.111452)
Supplement: Supplemental Files [file NIHMS1804143-supplement-Supplemental_Files.pdf]

Screening of SIRT6 inhibitors and activators: A novel activator has an impact on breast cancer cells

Jonna Tenhunen[a], Tomáš Kučera[b], Marjo Huovinen[a], Jenni Küblbeck[a], Egils Bisenieks[c], Brigita Vigante[c], Zaiga Ogle[c], Gunars Duburs[c], Martin Doležal[b], Ruin Moaddel[d], Maija Lahtela-Kakkonen[a,]*, Minna Rahnasto-Rilla[a,]*

[a]School of Pharmacy, University of Eastern Finland, Kuopio, Finland

[b]Faculty of Pharmacy in Hradec Králové, Charles University, Prague, Czech Republic

[c]Latvian Institute of Organic Synthesis, Riga, Latvia

[d]Biomedical Research Center, National Institute on Aging, National Institutes of Health, Baltimore, Maryland, United States

*Corresponding authors at: School of Pharmacy, University of Eastern Finland, Kuopio, Finland. E-mail address: minna.rahnasto@uef.fi (M. Rahnasto-Rilla), maija.lahtela-kakkonen@uef.fi (M. Lahtela-Kakkonen)

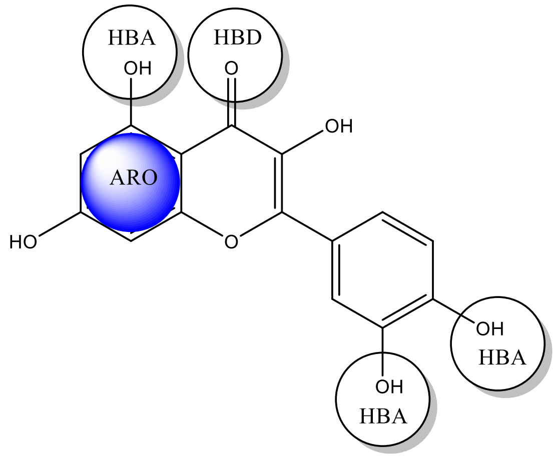

Figure S1: Pharmacophore model for virtual screening. ARO represents aromatic feature, HBA hydrogen bonding acceptor and HBD is hydrogen bonding donor feature.

Table S1. Cell culture conditions.

Cell line	Cells/well	Cell culture medium
MCF10A	150 000	DMEM/F12, 2% HS[a], 0.5 µg/ml, 1% P/S[b], 20 ng/ml EGF[c], Hydrocortisone, 100 ng/ml Cholera Toxin, 10 µg/ml Insulin
T47D	150 000	RPMI1640, 10% FBS[d], 1% P/S, 1% L-glut, 1 mM Na-pyruvate, 10 mM Hepes
MCF7	50 000	DMEM 11880, 10% FBS, 1% P/S, 1% L-glut[e]
ZR-75-1	50 000<br>500 000 (cell cycle analysis)	RPMI1640, 10% FBS, 1% P/S, 1% L-glut, 1 mM Na-pyruvate, 10 mM Hepes
SKBR3	75 000	RPMI1640, 10% FBS, 1% P/S, 1% L-glut + 1mM Na-pyruvate, 20 mM Hepes
MDA-MB-468	75 000<br>500 000 (cell cycle analysis)	DMEM 11880, 10% FBS, 1% P/S, 1% L-glut, 1 mM Na-pyruvate, 10 mM Hepes
HCC-1937	150 000<br>700 000 (cell cycle analysis)	RPMI1640, 10% FBS, 1% P/S, 1% L-glut
MDA-MB-231	50 000<br>500 000 (cell cycle analysis)	DMEM 11880, 10% FBS, 1% P/S, 1% L-glut, 1 mM Na-pyruvate, 10 mM Hepes
HS578T	50 000<br>500 000 (cell cycle analysis)	RPMI1640, 10% FBS, 1% P/S, 1% L-glut, 0.01 mg/ml Insulin

[a] Horse Serum, New Zealand origin, Standard (Sterile-Filtered) (Gibco); [b] Penicillin/Streptomycin (EuroClone);[c] Human Epidermal Growth Factor (Corning) [d] Fetal bovine serum (Life Technologies); [e] L-Glutamine (Biowest)

Table S2. Trivial names and references for synthesis of 1,4-DHP derivatives.

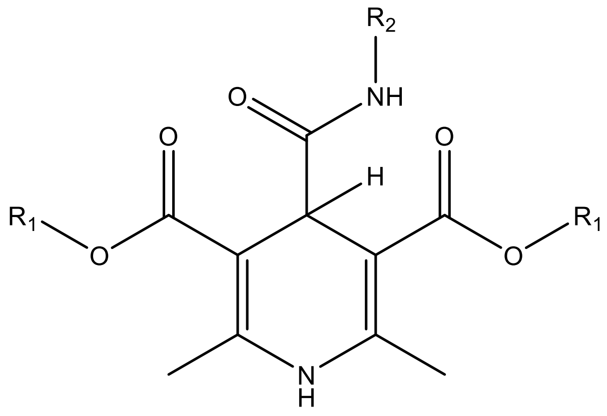

No	Name	R_1	R_2	Ref
7	Aspapyrone	C_2H_5	$CH(CO_2Na)CH_2CO_2Na$	M
8	Methapyrone	CH_3	$CH(CO_2Na)CH_2CH_2SCH_3$	M
9	Glutapyrone	C_2H_5	$CH(CO_2Na)C_2H_4CO_2Na$	Pat US 4485239A 1982
10	Proptauropyrone	C_3H_7	$(CH_2)_2SO_3Na$	M

M - New compound

Pat US - Biseniex, E.A., Dubur, G.J., Uldrikis, J.R. Veveris, M.M., Kimenis, A.A., Ivanov E.V. 2-(2,6-Dimethyl-3,5-diethoxycarbonyl-1,4-dihydropyridine-4-carboxamide glutaric acid, its disodium salt, and method of their preparation. Priority1980-06-12; Publication 1982-09-28; Pat US 4485239A.

(Comp.7) 3-(3,5-Bis(ethoxycarbonyl)-2,6-dimethyl-1,4-dihydropyridine-4-carboxamido)-3-carboxypropanoate sodium salt.

Mp: 224 ^{0}C (decomp.). ^{1}H NMR (400 MHz, DMSO-d_6): 8.99 (s, 1H), 7.16 (d, J = 5.2 Hz, 1H), 4.42 (s, 1H), 4.20-3.95 (m, 4H), 3.68-3.44 (m, 2H), 3.23 (dd, J = 9.5,7.5 Hz, 1H), 2.21 (s, 6H), 0.90 (t, J = 7.4 Hz, 6H). MS (ES+), m/z: 412 ($[M+H]^+$, 100). Calcl. for $C_{18}H_{23}N_2O_9Na$: C, 49.77; H, 5.34; N, 6.45; found: C, 48.38; H, 5.42; N, 6.31.

(Comp.8) (3,5-Bis(methoxycarbonyl)-2,6–dimethyl-1,4-dihydropyridine-4-carbonyl)methionine.

Mp: 159-160 ^{0}C. ^{1}H NMR (400 MHz, DMSO-d_6): 8.80 (s, 1H), 7.17 (d, J = 8.1 Hz, 1H), 4.40 (s, 1H), 4.16-4.10 (m, 1H), 3.61 (s, 6H), 2.36-2.21 (m, 2H), 2.22 (s, 6H), 2.02 (s,3H), 2.05-1.75 (m, 2H). MS (ES+), m/z: 401 ($[M+H]^+$, 100). Calcl. for $C_{17}H_{24}N_2O_7S$: C, 50.99; H, 6.04; N, 7.00; found: C, 50.76; H, 6.10; N, 6.87.

(Comp.9) Glutapyrone . Biseniex, E.A., Dubur, G.J., Uldrikis, J.R. Veveris, M.M., Kimenis, A.A., Ivanov E.V. 2-(2,6-Dimethyl-3,5-diethoxycarbonyl-1,4-dihydropyridine-4-carboxamide glutaric acid, its disodium salt, and method of their preparation. Priority1980-06-12; Publication 1982-09-28; Pat US 4485239A;

(Comp.10) 2-(2,6-Dimethyl-3,5-bis(propoxycarbonyl)-1,4-dihydropyridine-4-carboxamido)ethane-1-sulfinate sodium salt.

Mp: 148-150 ^{0}C. ^{1}H NMR (400 MHz, DMSO-d_6): 8.82 (s, 1H), 7.29 (t, J = 5.4 Hz, 1H), 4.38 (s, 1H), 4.09-3.89 (m, 4H), 3.23(q, J = 7.6 Hz, 2H), 2.47-2.39 (m, 2H), 2.21 (s, 6H), 1.66-1.51 (m, 4H), 0.90 (t, J = 7.4 Hz, 6H). MS (ES+), *m/z*: 416 ([M+H]$^+$, 100). Calcl. for $C_{18}H_{27}N_2O_7SNa$: C, 49.25; H, 6.20; N, 6.38; found: C, 48.98; H, 6.30; N, 6.22.

Table S3. SIRT6 activity data of compound 1 analogs. The data is presented as means, n=2.

Subtrate concentration	70 µM	200 µM
	Fold change in SIRT6 deacetylase activity	
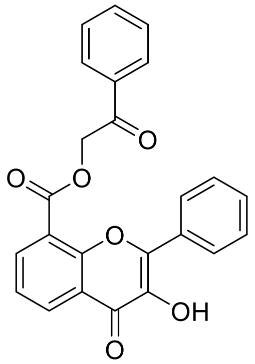	1.4±0.4	1.2±0.2
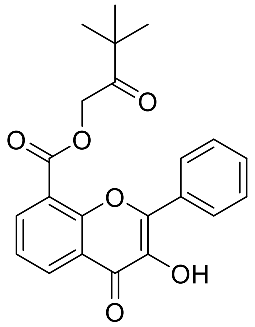	0.8 ±0.1	0.9±0.3
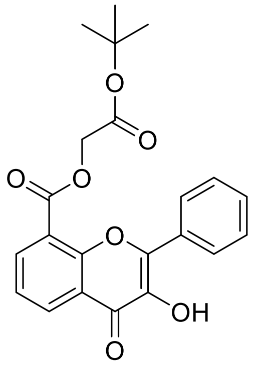	0.7±0.1	0.9±0.3
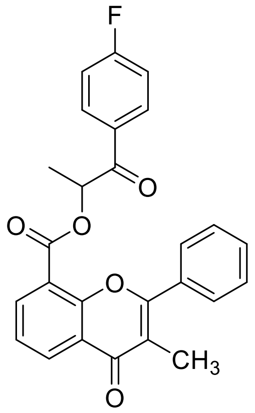	1.2±1.3	1.0±0.1

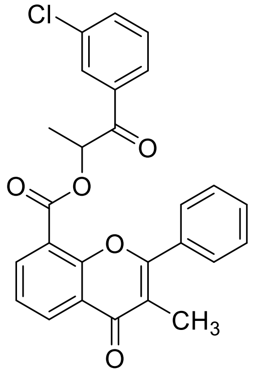	1.2±0.2	1.1±0.1
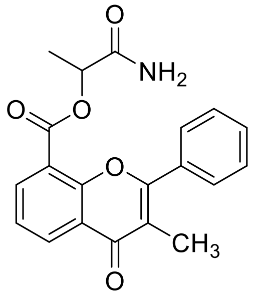	0.8±0.5	0.9±0.4
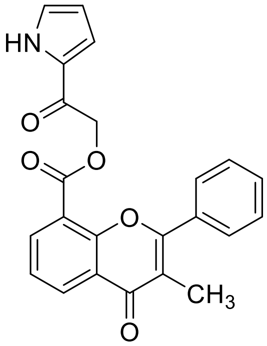	1.2±0.1	1.2±0.2

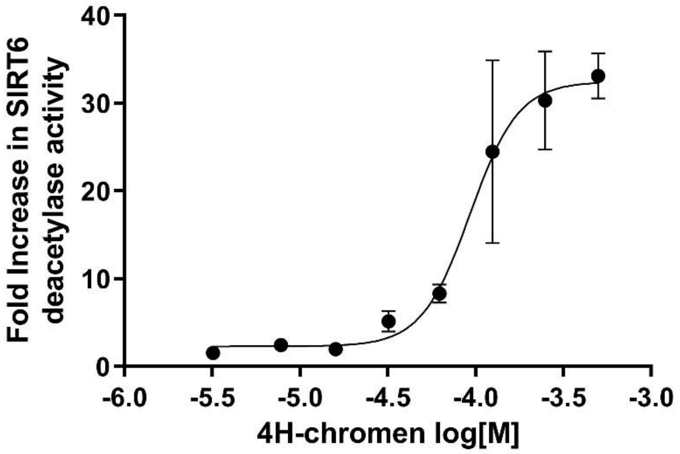

Figure S2. 4H-chromen dose-dependently induces SIRT6 activity. Dose response effect on SIRT6 deacetylation activity by 4H-chromen by HPLC assay. The data are presented as mean ± SEM (n=3).

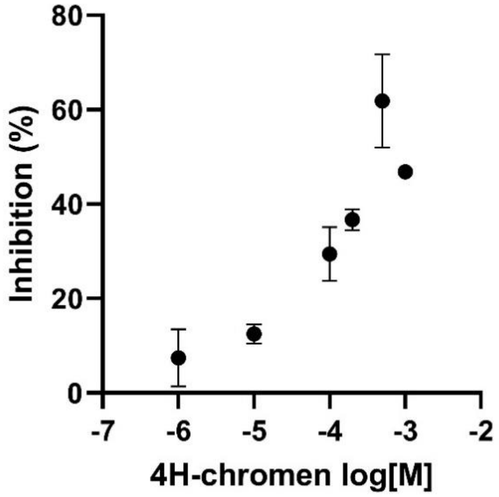

Figure S3. 4H-chromen slightly inhibits SIRT6 demyristoylase activity. Dose response effect on SIRT6 demyristoylase activity by 4H-chromen. The data are presented as mean ± SEM (n=3).

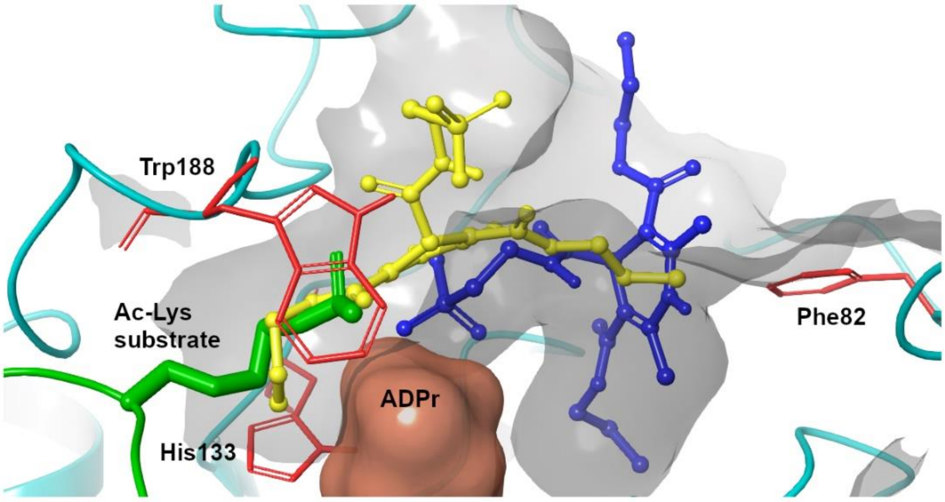

Figure S4. SIRT6 inhibiting dihydropyridines showed two alternative poses in docking studies. The dihydropyridines could occupy the substrate binding site in one pose (yellow compound) and in other pose the C-pocket (blue compound). Compound 10 was used to represent the poses.

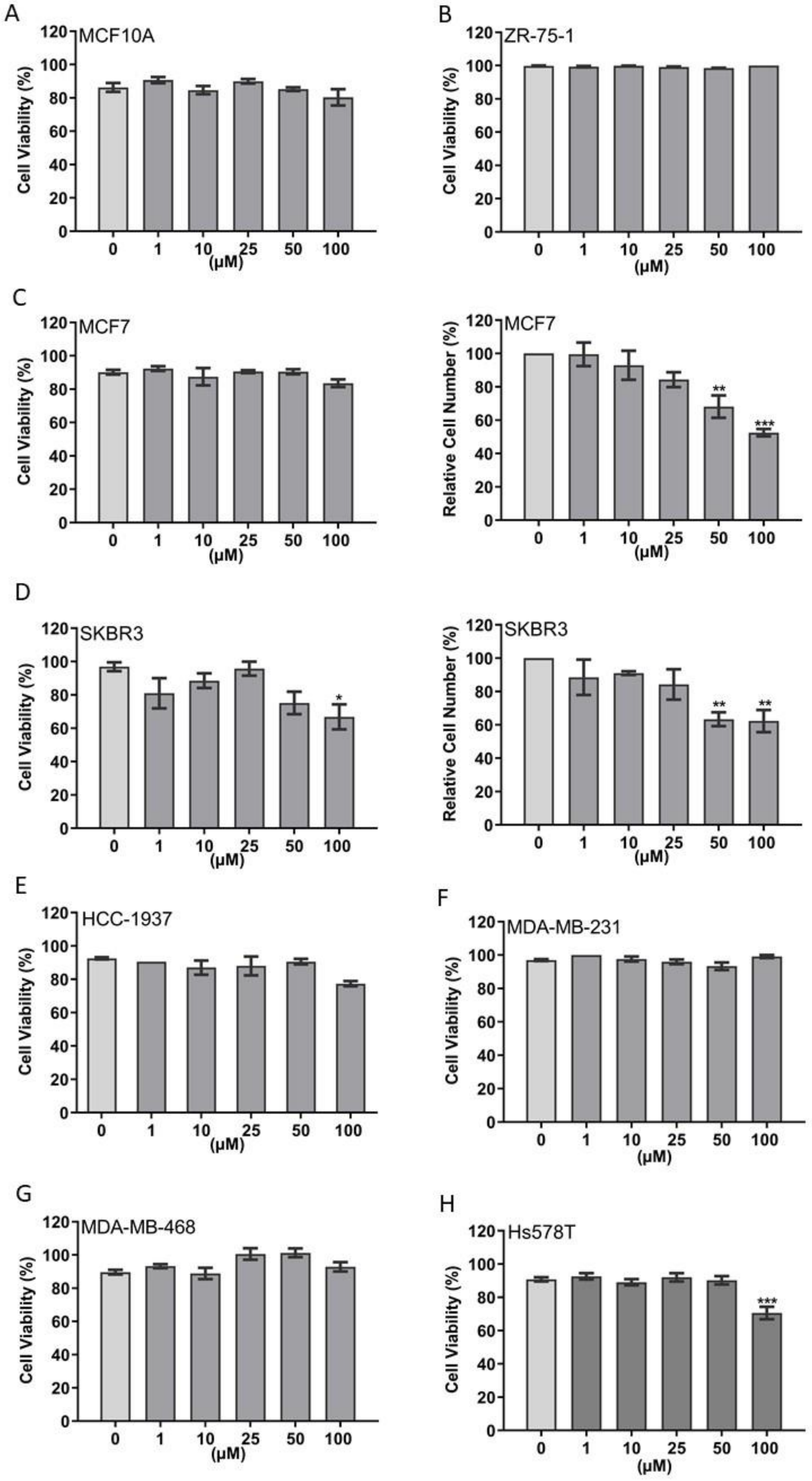

Figure S5. Cell vialibility and cell number studies of 4-chromen in different breast cancer cell lines. Data represent the mean ± SEM of three experiments, and the statistical analysis was carried out with one-way ANOVA with Dunnett’s post hoc test by comparing treated groups (gray bars) to DMSO control groups (light gray bars).
